# The exercise–gut–muscle axis in physical frailty: A narrative review

**DOI:** 10.1016/j.jarlif.2026.100087

**Published:** 2026-09-23

**Authors:** Shinichiro Morishita, Naoya Iida, Toshimi Sato, Yasuhiro Endo, Daiki Tanno, Shoji Yabuki

**Affiliations:** a Department of Physical Therapy, School of Health Sciences, Fukushima Medical University, Fukushima, Japan; b Department of Clinical Laboratory Sciences, School of Health Sciences, Fukushima Medical University, Fukushima, Japan

**Keywords:** Gut microbiome, Exercise, Skeletal muscle, Physical frailty, Aging, Gut–muscle axis

## Abstract

The prevalence of physical frailty has increased in recent years, owing to an aging population. This geriatric syndrome is characterized by a decline in physiological reserve and physical function. Thus, the risk of adverse health outcomes amongst individuals in a state of frailty is high. Frailty development is strongly influenced by skeletal muscle dysfunction, which involves reductions in muscle mass, strength, and metabolic capacity. Exercise is the most effective approach for the maintenance of muscle function and prevention of functional decline in older adults. However, the biological mechanisms underlying its role in aging remain unclear. The gut microbiota produces various bioactive metabolites that affect metabolic regulation, immune responses, and inflammatory pathways. Exercise may modify the composition and metabolic function of the gut microbiota in some studies, although the findings remain inconsistent. These exercise-associated alterations may influence the production of microbial metabolites that may affect skeletal muscle metabolism. Recent evidence on age-related alterations in the gut microbiota is reviewed here, with a focus on how these changes relate to skeletal muscle physiology. Furthermore, the mechanisms by which exercise modifies the composition and metabolic pathways of the gut microbiota are described, highlighting the concept of the “exercise–gut–muscle axis” as a framework linking physical activity, the gut microbiome, skeletal muscle, and physical frailty trajectories. Our narrative synthesis integrates the emerging evidence on exercise-microbiome-muscle interactions. This may help identify priorities for future research on lifestyle-based strategies that support healthy aging and may help delay physical frailty in older adults.

## 1 Introduction

Improvements in public health, medical care, and living environments have markedly increased the average life expectancy worldwide, resulting in a progressively aging population [1,2]. Although life expectancy has continued to rise, age-related diseases that compromise independence and quality of life have become increasingly common, making frailty a major health concern [3,4].

Frailty is characterized by a decline in physiological reserve across multiple organ systems [3,4]. Individuals with frailty are vulnerable to stressors, leading to an increased risk of falls, functional impairment, and death [[3], [4], [5], [6]]. A key biological factor underlying frailty is sarcopenia, which involves the progressive deterioration of skeletal muscle mass and function during aging. Sarcopenia involves a gradual loss of muscle mass, strength, and physical performance over time [5]. Given the central role of skeletal muscle in physical function and metabolic health, muscle health must be maintained to sustain a healthy life [5,6]. Sarcopenia is considered primary (or age-related) when aging is the sole apparent cause, or secondary when additional factors—physical inactivity, inadequate energy or protein intake, or chronic disease—contribute. However, particularly in older adults, these etiological factors frequently coexist, blurring the distinction [5].

Regular exercise is considered a highly effective non-pharmacological strategy for preserving skeletal muscle function during aging [[7], [8], [9], [10]]. In older adults, both aerobic and resistance exercise have been shown to enhance muscle strength, physical performance, and metabolic health [[7], [8], [9]]. Regular physical activity is also associated with improved mitochondrial function, enhanced insulin sensitivity, increased muscle protein metabolism, and reduced chronic inflammation [9,10]. Accordingly, exercise plays a central role in reducing frailty risk and preserving physical independence among older adults [[6], [7], [8]].

Despite abundant clinical evidence of the benefits of exercise, the biological mechanisms through which physical activity promotes health in older adults remain unclear. Recent evidence suggests that the gut microbiome may serve as an important link between lifestyle factors and host physiological function [[11], [12], [13]]. The gut microbiome consists of trillions of microorganisms, including bacteria, viruses, and fungi [11,12]. These microorganisms influence host physiology through complex metabolic and signaling pathways; they play crucial roles in nutrient metabolism, immune regulation, and energy homeostasis [12,13].

Human cohort studies have linked age-related alterations in the gut microbiome to frailty, reduced physical function, and poorer overall health in older populations [[14], [15], [16]]. Large cohort studies have demonstrated that variations in gut microbiota composition are closely linked to dietary patterns and overall health status in older adults [14]. Jackson et al. (2016) further reported microbial signatures associated with early frailty [15]. Among community-dwelling older adults, physical fitness has been associated with dietary patterns, gut microbial composition, and metabolomic profiles [16]. These observations suggest that the gut microbiome may be associated with physical function and frailty during aging, although causal relationships remain unclear.

One plausible mechanism involves microbial metabolites. Gut bacteria produce numerous biologically active metabolites, such as short-chain fatty acids (SCFAs), bile acid metabolites, indole-derived compounds, and products of amino acid metabolism [[17], [18], [19], [20], [21]]. SCFAs such as acetate, propionate, and butyrate have considerable effects on inflammation, mitochondrial metabolism, glucose homeostasis, and insulin sensitivity [[17], [18], [19]].

The concept “gut–muscle axis” has been coined based on previous studies. This term describes the interaction between the gut microbiota and the metabolism and function of skeletal muscle [[22], [23], [24], [25]]. Importantly, exercise has been shown to influence the microbial composition of the gut as well as its metabolic function [[26], [27], [28], [29], [30], [31], [32], [33], [34], [35], [36], [37], [38]]. Animal and human studies indicates that habitual physical activity may enhance microbial diversity in certain settings and enriches bacterial taxa associated with beneficial metabolic functions, including SCFA production [27,28,30,31,38].

Earlier reviews have highlighted links between the gut microbiome and skeletal muscle physiology, and have also addressed how exercise shapes gut microbial composition. However, only a few reviews have integrated these findings into a unified framework linking physical activity, microbiome-mediated pathways, skeletal muscle physiology, and frailty prevention in aging. Therefore, a narrative synthesis focused on the exercise–gut–muscle axis is needed to clarify how physical activity promotes healthy aging beyond its direct effects on skeletal muscle [25,39].

Despite growing interest in the relationship among physical activity, the gut microbiota, and skeletal muscle physiology, critical gaps remain in our understanding of how these factors interact to influence aging-related outcome. Previous reviews have focused either on exercise-induced changes in the gut microbiota or on the gut-muscle axis. There are few unified frameworks that integrate these findings to explain how physical activity influences age-related muscle function and the progression of physical frailty through gut microbiota-mediated pathways. Therefore, the central question addressed in this article is how exercise-induced modulation of the gut microbiota influences age-related skeletal muscle function, physical performance, and the progression of physical frailty.

To this end, we propose the “exercise–gut–muscle axis” as a conceptual framework linking physical activity, regulation of the gut microbiota, production of microbial metabolites, skeletal muscle physiology, and the progression of physical frailty. Furthermore, we examine the biological mechanisms underlying this framework, evaluate the current evidence, and identify key limitations. We outline directions for future research necessary to translate this concept into strategies for healthy aging and physical frailty prevention. Importantly, this framework is consistent with the newly proposed “trajectory-based model of aging” by focusing not only on frailty as a clinical endpoint but also on the biological and functional pathways that may influence the progression of frailty over time. We believe that a narrative review is the most appropriate article type because the aim of this article is to synthesize current evidence on the interactions among physical activity, the gut microbiome, skeletal muscle physiology, and frailty, and to develop a conceptual framework linking these processes. Most available evidence is associative, and studies have rarely distinguished primary from secondary sarcopenia. The exercise–gut–muscle axis requires validation through longitudinal and intervention studies and applies specifically to physical frailty rather than psychological, social, or comprehensive frailty.

Accordingly, this article proceeds through three stages. First, synthesizing human and preclinical evidence on exercise, the gut microbiota, skeletal muscle, and physical frailty, distinguishing associative from mechanistic findings. Second, proposing a mechanistic exercise–gut–muscle framework while identifying its hypothetical components. Third, discussing translational implications and priorities. The framework should therefore be interpreted as hypothesis-generating rather than as established causal mechanisms. The principal contribution is integrative and heuristic, unifying adjacent literature within a conceptual framework rather than presenting new evidence or practice-changing recommendations.

## 2 Literature search strategy

This narrative review aimed to develop a conceptual framework linking physical activity, the gut microbiome, skeletal muscle physiology, and frailty trajectories during aging.

A targeted, non-systematic PubMed search was conducted in May 2026 using combinations of the keywords: “exercise,” “physical activity,” “gut microbiota,” “gut microbiome,” “skeletal muscle,” “muscle function,” “frailty,” “sarcopenia,” and “aging.” Original research articles, including randomized controlled trials, observational studies, and other clinical and translational studies, were considered.

Studies were selected based on their relevance to one or more components of the exercise–gut–muscle axis. Original reports were preferentially cited. Previous reviews were used primarily to summarize the broader evidence base and provide contextual interpretation, and their included primary studies were not treated as independent additional evidence.

## 3 Aging and the gut microbiome

The composition and function of the gut microbiome change considerably across the lifespan. Factors such as delivery mode, diet, antibiotic exposure, and environmental contact strongly affect microbial colonization during infancy [[40], [41], [42]]. Large population-based studies across age groups have revealed that gut microbial composition varies based on age and geography and continues to evolve throughout the human lifespan [40]. The gut microbiome generally stabilizes as individuals mature; it supports metabolic and immune homeostasis during adulthood [12,41,43]. However, this stability may gradually decline with age.

Aging is accompanied by changes in both the composition and functional characteristics of the gut microbiome [[14], [15], [16],^42^,^44^]. In older adults, gut microbial composition is influenced by dietary patterns, living environment, and overall health status [14]. Odamaki et al. [42] reported age-related changes in gut microbiota composition across a wide age range, from newborns to centenarians. Furthermore, studies on longevity have reported characteristic changes in microbial communities with aging [44]. While the extent and direction of reported changes differed among studies and populations, overall findings indicate that gut microbial profiles in older adults are distinct from those observed in younger individuals.

Aging is often associated with reduced microbial diversity, although this is not consistent. Moreover, aging may depend on health status, medication use, dietary patterns, and living environment [[14], [15], [16],[44], [45], [46]]. Reduced diversity, a marker of microbiome resilience [45,46], was associated with physiological vulnerability, frailty, and unfavorable health profiles in older cohorts [14,15].

In addition to reduced diversity, aging is frequently accompanied by shifts in specific microbial taxa. The abundance of beneficial bacteria, such as *Faecalibacterium* and *Bifidobacterium,* may decrease with age, whereas microorganisms associated with dysbiosis or inflammation may become more prevalent [14,42,[44], [45], [46]]. These alterations in the gut microbiome affect gut barrier integrity, immune regulation, and microbial metabolite production.

One important consequence of age-related microbiome alteration is chronic low-grade inflammation. Aging is often accompanied by a persistent inflammatory state referred to as inflammaging [2,47]. This may be involved in many age-related diseases, including metabolic dysfunction, cardiovascular disease, neurodegenerative diseases, and frailty [2,47]. Given the central role of the gut microbiome in immune regulation, changes in microbial composition during aging may contribute to systemic inflammatory signaling [15,16,[47], [48], [49]].

The gut microbiome also produces microbial metabolites, including SCFAs. SCFAs are produced through bacterial fermentation of dietary fiber. In addition to serving as energy sources within the gut, SCFAs function as signaling molecules that influence glucose and lipid metabolism, intestinal barrier function, and immune regulation [[17], [18], [19]]. These pathways are highly important, as they facilitate intact metabolic signaling, mitochondrial function, and a balanced inflammatory environment for skeletal muscle maintenance and repair.

Beyond SCFAs, gut microorganisms produce a variety of metabolites, including bile acid derivatives, indole-based compounds, and amino acid–derived metabolites, which influence host receptor signaling and downstream physiological pathways [20,21,43]. These metabolites may affect insulin sensitivity, oxidative stress, mitochondrial function, and systemic metabolic homeostasis. Therefore, they are relevant to skeletal muscle physiology.

The gut microbiome is associated with physical performance in older adults. Jackson et al. (2016) identified microbiological signatures associated with early frailty [15]. Similarly, Castro-Mejia et al. [16] reported associations between physical fitness in community-dwelling older adults and both gut microbial characteristics and metabolomic profiles. Recent meta-analyses have further shown that gut microbial profiles differ between older adults with sarcopenia and those without the condition [50]. In primary sarcopenia, age-associated gut dysbiosis may interact with chronic inflammation, anabolic resistance, and mitochondrial dysfunction to promote muscle deterioration [22,51,52]. In secondary sarcopenia, disease-specific inflammation, inactivity, and malnutrition may further modify the gut microbiota and muscle [22,51,53]. Thus, the exercise–gut–muscle axis may be hypothesized to involve partially shared downstream pathways across both forms of sarcopenia, although their upstream drivers and the relative contribution of the gut microbiota may differ. The available systematic reviews did not report studies directly comparing gut microbiota profiles or exercise-related microbiome responses between clearly defined primary and secondary sarcopenia populations [51,54].

Overall, age-related gut microbiome shifts may be linked to skeletal muscle physiology through metabolic, inflammatory, and immunological pathways (Fig. 2).

## 4 The gut–muscle axis

The term “gut–muscle axis” describes interactions between intestinal microorganisms and skeletal muscle metabolism and function [[22], [23], [24], [25]]. This concept is especially relevant in aging because both the gut microbiome and skeletal muscle undergo significant age-related changes that may lead to physical frailty.

A central mechanism underlying the gut–muscle axis involves microbial metabolites. Gut bacteria metabolize dietary substrates, particularly dietary fiber, to produce SCFAs such as acetate, propionate, and butyrate [[17], [18], [19]]. These metabolites can enter the systemic circulation and affect tissues beyond the gut, including skeletal muscle. These are all critical for skeletal muscle physiology.

The SCFA butyrate has a crucial role in mitochondrial function and oxidative metabolism [[17], [18], [19],^55^]. Mitochondrial dysfunction is a major characteristic of muscle tissue associated with aging. Microbial metabolites affect the health of muscle mitochondria under various exercise conditions [55]. Thus, microbial metabolite signaling may represent one pathway through which the gut microbiome affects muscle endurance, metabolic efficiency, and adaptability.

The gut microbiome shapes host immunity, and microbial products can affect both local intestinal immune responses and systemic inflammatory tone [48,49]. This is particularly relevant in older adults because chronic low-grade inflammation contributes to muscle wasting, anabolic resistance, and functional decline [2,47]. Microbiome-mediated immune regulation may affect skeletal muscle indirectly through inflammatory pathways. Furthermore, changes in the gut microbiota may promote age-related decline in muscle maintenance, which is characterized by a blunted muscle protein synthetic response to dietary protein intake and exercise [56].

Experimental data support a functional connection between gut microbes and muscle physiology. In germ-free mice, the absence of a gut microbiome was associated with reduced skeletal muscle mass and diminished muscle function, both of which were partially recovered following microbiota reconstitution [57]. This study provides important empirical evidence that gut microbiota can affect skeletal muscle [57].

Although evidence in humans is limited, several previous studies suggest that microbiome characteristics are associated with fitness function. For example, Estaki et al. [26] demonstrated that individuals with higher cardiorespiratory fitness exhibit greater gut microbial diversity and distinct metagenomic profiles. Barton et al. (2018) revealed that microbial communities in professional athletes differed from those in more sedentary individuals, particularly at the functional metabolic level [28]. Moreover, Scheiman et al. [58] identified a performance-enhancing microbe linked to lactate metabolism in elite athletes. Although studies on athletes cannot be directly generalized to older adults, they support the hypothesis that the composition and function of the microbiome are linked to athletic performance and physical function.

Recent human and translational studies support this field. Ahn et al. [59] used human fecal microbiota transplantation to identify intestinal microorganisms that enhance skeletal muscle strength in mice. Furthermore, recent systematic reviews concluded that the gut microbiome is plausibly linked to muscle mass, strength, and sarcopenia-related outcomes [50,51,60]. Thus, although causality in humans remains incompletely established, the cumulative evidence supports the biological plausibility of the gut–muscle axis.

Collectively, these findings indicate that the gut microbiome may affect skeletal muscle through microbial metabolite production, immune regulation, and metabolic signaling pathways. These interactions provide a conceptual basis for understanding how microbiome alterations may contribute to age-related decreases in muscle function. Representative experimental studies examining microbiome–muscle interactions are summarized in Table 1.

**Table 1:** Proposed biological mechanisms linking the gut microbiome to skeletal muscle function.

Mechanism / factor	Main biological relationship to skeletal muscle	Key findings	Main evidence type	Representative references (Author, Year)
Short-chain fatty acids (SCFAs): acetate, propionate, butyrate	May affect skeletal muscle through improved insulin sensitivity, substrate metabolism, mitochondrial function, and reduced inflammatory signaling	SCFAs are major microbial metabolites produced from dietary fiber fermentation; they are involved in host metabolic regulation and immune homeostasis	Mechanistic reviews, translational evidence	Koh et al. [17]; Canfora et al. [18]; Dalile et al. [19]
Butyrate and mitochondrial function	May support mitochondrial oxidative metabolism and muscle metabolic efficiency	Butyrate is a metabolite potentially associated with mitochondrial health, which is important because mitochondrial dysfunction is a hallmark of muscle aging	Mechanistic review	Koh et al. [17], 2016; Canfora et al. [18], 2015; Dalile et al. [19]; Xie & Huang [55]
Microbiome-mediated immune regulation	May indirectly affect muscle mass and function through regulation of chronic low-grade inflammation	The gut microbiome regulates systemic immune tone; chronic inflammation leads to anabolic resistance, muscle loss, and frailty	Basic and conceptual evidence	Belkaid & Hand [49]; Lozupone et al. [48]; Ferrucci and Fabbri [47]
Gut barrier and metabolic homeostasis	May help maintain a physiological environment favorable to muscle health	Microbial metabolites function in gut barrier integrity and metabolic homeostasis affecting systemic physiology	Mechanistic reviews	Koh et al. [17]; Canfora et al. [18]; Dalile et al. [19]; Agus et al. [20]; Nicholson et al. [21]
Experimental evidence from germ-free models	Supports a direct functional link between the gut microbiome and muscle phenotype	Germ-free mice exhibit reduced skeletal muscle mass and impaired muscle function; microbiota reconstitution partially restored these abnormalities	Animal experiment	Lahiri et al. [57]
Microorganisms associated with muscle strength	Suggests that specific intestinal microbes are associated with skeletal muscle strength and muscle-related phenotypes.	Translational studies using human-derived microbiota in mice identified intestinal microorganisms associated with skeletal muscle strength.	Translational microbiota transplantation study (human-derived microbiota in mice)	Ahn et al. [59]
Urolithin A as a microbiome-derived metabolite important for muscle strength	Supports the concept that microbiome-derived metabolites can affect muscle-related outcomes in humans	Randomized clinical trials revealed improvements in muscle strength , exercise performance, and mitochondrial biomarkers	Human randomized trials	Singh et al. [64]
Gut microbiota and sarcopenia framework	Conceptual model linking microbiome alterations with muscle mass decline and frailty	Reviews propose that microbiota-driven inflammation, metabolite production, and anabolic resistance lead to sarcopenia	Conceptual / narrative review	Grosicki et al. [25]
Microbiome role in anabolic resistance	Potential effect of microbiota on muscle protein metabolism during aging	Evidence suggests that microbiome alterations lead to age-related anabolic resistance and sarcopenia mechanisms	Systematic / mechanistic review	Watson et al. [56]

## 5 Exercise-induced changes in the gut microbiome

Exercise is widely recognized as a key lifestyle strategy for maintaining health with aging. Exercise may, at least in part, induce changes in the gut microbiota [[26], [27], [28], [29], [30], [31], [32], [33], [34], [35], [36], [37], [38],^61^].

Several observational studies revealed distinct gut microbiome characteristics between individuals with higher levels of physical activity or physical fitness and sedentary individuals. Greater cardiorespiratory fitness was linked to increased gut microbial diversity and unique metagenomic functional profiles, independent of several confounding variables [26]. Moreover, Bressa et al. [31] reported differences in gut microbiota profiles between active and sedentary women. Similarly, microbial communities in professional athletes differed from those in sedentary controls, especially at the functional metabolic level [28]. Taken together, these findings suggest that the gut microbial ecosystems of individuals who engage in regular physical activity or possess high physical fitness differ from those of individuals who do not.

Intervention studies further suggest that exercise can alter microbiome composition. In previously sedentary adults, Allen et al. [27] showed that six weeks of aerobic training modified both the composition and functional profile of the gut microbiome. The extent and direction of the microbial response differed between lean and obese individuals. These findings imply that the host’s metabolic condition may influence how the gut microbiome responds to exercise. Similarly, Munukka et al. [30] reported that six weeks of endurance training alters the gut metagenome in overweight women. In a Japanese study of healthy older women, brisk walking-based aerobic exercise increased intestinal *Bacteroides*, suggesting that feasible community-based exercise affects the microbiome in older adults [38].

Animal studies have produced similar observations. In a mouse model, Allen et al. [33] showed that voluntary and forced exercise exert differential effects on gut microbial composition. These findings demonstrate that, under controlled experimental conditions in mice, physical activity can shape the gut microbial environment; however, their applicability to humans remains uncertain.

Exercise-induced alterations in the gut microbiome may enhance SCFA production and promote the enrichment of microbial taxa associated with beneficial metabolic functions [26,28,58]. Furthermore, SCFAs regulate metabolic and immune pathways associated with muscle health [[17], [18], [19]]. This could serve as a biologically plausible link connecting exercise, microbial ecology, and host muscle physiology. However, findings across studies are not entirely consistent, and not all exercise interventions result in the same microbiome changes [27,29,30,33].

Several physiological mechanisms may explain the mechanisms through which exercise affects the intestinal environment. Physical activity may alter gut transit time, intestinal perfusion, bile acid metabolism, substrate availability, immune signaling, and barrier function [32,62,63]. Additionally, exercise may reduce systemic inflammation and improve metabolic regulation [7,9]. These effects may further support a healthy gut ecosystem.

Overall, evidence from recent systematic reviews and meta-analyses suggests a link between physical activity and changes in the gut microbiome, although heterogeneity remains [34,35,37]. From a mechanistic perspective, exercise is not only a muscle-directed stimulus but may also modify the intestinal environment and its metabolic output.

This observation is particularly relevant to aging, as older adults generally engage in lower levels of physical activity. Additionally, they present with altered gut microbial composition and increased inflammatory burden. Therefore, exercise has established direct benefits for skeletal muscle function and may also influence host physiology through microbiome-related pathways, although the latter remains hypothetical (Table 2).

**Table 2:** Human studies examining the effects of exercise on the gut microbiome.

Study	Study design	Population / Model	*n*	Main microbiome finding
Clarke et al. [61]	Cross-sectional (athlete cohort)	Professional rugby players vs. controls	86	Professional athletes exhibit increased gut microbial diversity and enrichment of taxa associated with metabolic health.
Estaki et al. [26]	Cross-sectional	Healthy adults with varying levels of cardiorespiratory fitness	39	Cardiorespiratory fitness is positively associated with microbial diversity and distinct microbial composition.
Bressa et al. [31]	Cross-sectional	Active vs. sedentary women	40	Active women show higher abundance of beneficial bacterial taxa than sedentary individuals.
Allen et al. [27]	Exercise intervention	Lean and obese adults	32	Exercise alters gut microbiome composition and functional pathways related to SCFA metabolism.
Munukka et al. [30]	Exercise intervention	Overweight women undergoing endurance training	17	Six-week endurance exercise modifies gut microbial composition and metabolic pathways.
Cronin et al. [29]	Exercise intervention + nutrition	Physically active adults	90	Exercise and protein supplementation affect microbial functional pathways and metabolomic profiles.
Barton et al. [28]	Athlete cohort	Professional athletes vs. healthy controls	86	Athletes exhibit distinct gut microbiome composition compared with sedentary individuals.
Morita et al. [38]	Exercise intervention	Healthy elderly women (brisk walking program)	32	Aerobic exercise increases the relative abundance of *Bacteroides* in older adults.

## 6 Exercise–gut–muscle interactions and skeletal muscle function

In the previous sections, we have discussed the mechanisms underlying the effects of the gut microbiota on the skeletal muscle physiology. We also discussed how exercise reshapes the gut microbial environment. However, the biological link between these processes remains unclear. Exercise affects skeletal muscle through direct physiological adaptations and may additionally influence muscle-related outcomes through gut microbiota-mediated pathways. Herein, we refer to this framework as the “exercise–gut–muscle axis” (Fig. 1). A central mechanism linking the exercise–gut–muscle axis involves microbial metabolites, particularly SCFAs generated through bacterial fermentation of dietary fiber, which influence host metabolism and immune function [[17], [18], [19]]. Preclinical and mechanistic studies suggest that SCFAs may affect skeletal muscle by improving insulin sensitivity, regulating inflammatory pathways, and supporting mitochondrial function [[17], [18], [19],^55^]. As these processes are central to muscle metabolism and performance, microbial metabolites may represent biologically relevant intermediates linking gut microbes to the physiology of skeletal muscle.

**Fig. 1:**
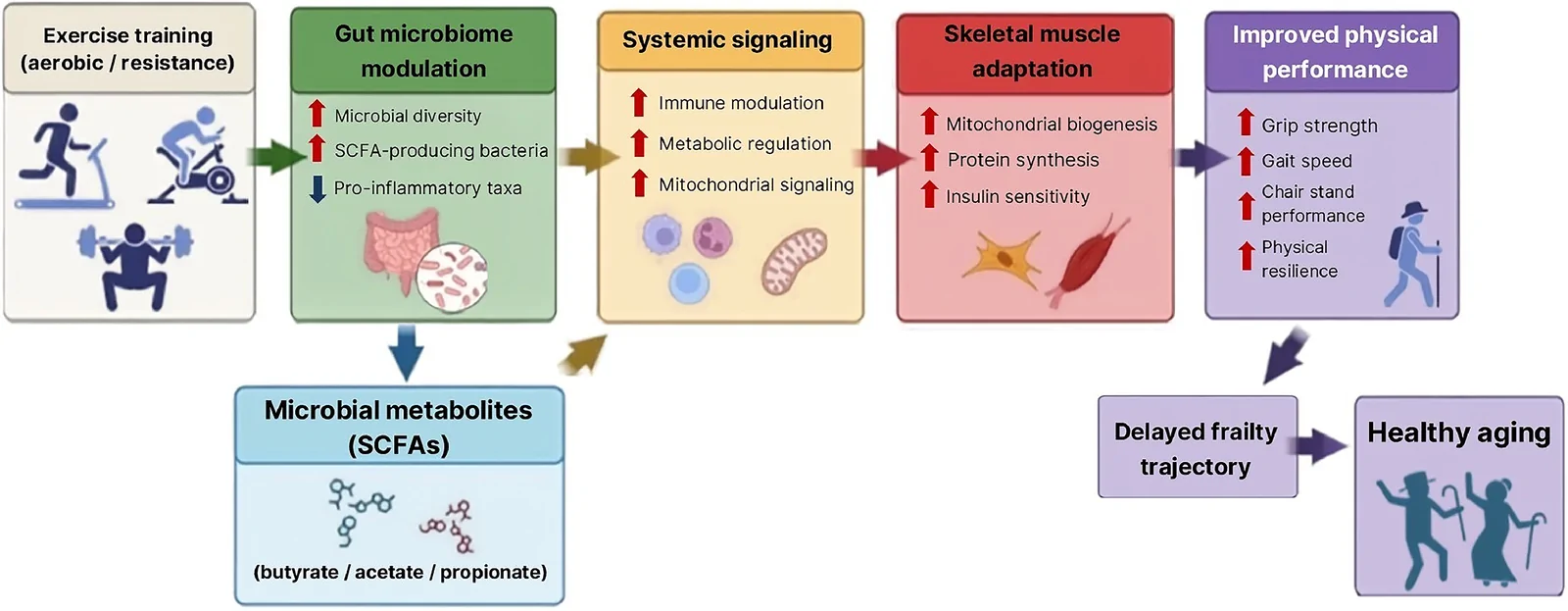
Proposed conceptual framework of the exercise–gut–muscle axis. Regular exercise, including aerobic and resistance training, may modify the gut microbiome, including potential changes in microbial diversity and the relative abundance of taxa involved in the production of metabolites such as short-chain fatty acids (SCFAs). These microbiome-derived metabolites affect systemic physiological pathways, including immune modulation, metabolic regulation, and mitochondrial signaling. Exercise-induced microbiome alterations may contribute to skeletal muscle adaptations such as enhanced mitochondrial biogenesis, improved protein synthesis, and increased insulin sensitivity through these mechanisms. Through these interconnected pathways, exercise-induced alterations in the gut microbiome may improve physical performance and contribute to delayed physical frailty trajectories and healthy aging. The arrows represent proposed or biologically plausible relationships and should not be interpreted as evidence of established causal mediation in humans. Created with BioRender.com.

**Fig. 2:**
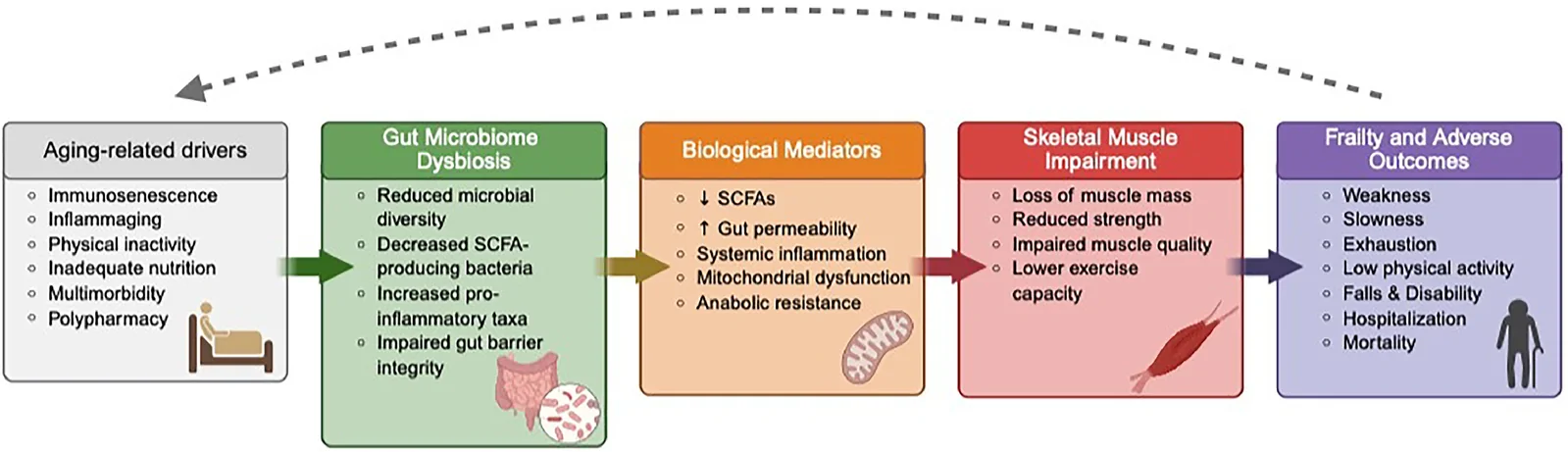
The pathway linking aging-related microbiome dysbiosis to skeletal muscle impairment and physical frailty. Aging is associated with multiple physiological and environmental factors, including immunosenescence, inflammaging, physical inactivity, inadequate nutrition, multimorbidity, and polypharmacy. These factors may be associated with gut microbiome dysbiosis characterized by reduced microbial diversity, decreased abundance of SCFA-producing bacteria, increased abundance of potentially pro-inflammatory taxa, and impaired integrity of the gut barrier. Microbiome alterations may affect skeletal muscle physiology through biological mediators such as reduced SCFA production, increased gut permeability, systemic inflammation, mitochondrial dysfunction, and anabolic resistance. These processes may lead to skeletal muscle impairment, including reduced muscle mass, decreased strength, and impaired muscle quality, ultimately contributing to physical frailty and adverse health outcomes such as falls, disability, hospitalization, and mortality. The proposed pathways are based on associative human evidence and mechanistic evidence derived partly from preclinical studies; causal relationships in humans remain unconfirmed. Created with BioRender.com.

Animal studies strongly support this hypothesis. Lahiri et al. (2019) demonstrated that germ-free mice lacking a gut microbiome exhibit reduced skeletal muscle mass and impaired muscle function. Furthermore, the muscle phenotype was partially restored by microbiota reconstitution [57]. A recent study identified gut microorganisms associated with skeletal muscle strength using human microbiota transplantation experiments in mice, further supporting the role of microbiome-related pathways in the regulation of muscle biology [59].

Exercise may reinforce this interaction by altering microbial composition and metabolic activity. Physical activity is associated with the enrichment of bacterial taxa capable of producing beneficial metabolites, such as SCFAs [[26], [27], [28],^30^,^38^,^58^]. These may affect energy utilization, inflammatory responses, and metabolic flexibility in skeletal muscle. Thus, exercise-induced microbiome changes may amplify or complement the direct physiological effects of exercise on muscle tissue.

Immune regulation may represent another important mechanism linking exercise, the microbiome, and skeletal muscle. Aging is associated with chronic low-grade inflammation, which leads to muscle loss and functional decline [2,47]. Exercise can reduce systemic inflammation and improve immune regulation [7,9], whereas the gut microbiome represents a major determinant of immune homeostasis [48,49]. Therefore, exercise-induced improvements in microbial balance may contribute to the creation of a physiological environment that enhances immune regulation and supports muscle maintenance.

Mitochondrial function is another plausible convergence point. Skeletal muscle heavily depends on mitochondria for ATP production during physical activity, and mitochondrial dysfunction is a hallmark of aging muscle. Recent studies suggest that exercise, particularly under different modes of activity, affects mitochondrial function in aged muscle [55]. Although the mechanisms remain unclear, they provide a plausible explanation for how microbiome-derived metabolites and exercise affect muscle quality.

Urolithin A, a metabolite generated by the gut microbiome from ellagitannins, has been shown to enhance muscle strength, exercise performance, muscle endurance, and biomarkers of mitochondrial function in middle-aged and older adults [64]. Additionally, it enhanced mitochondrial health through the activation of mitophagy and the improvement of mitochondrial function. This may contribute to improvements in muscle endurance and metabolic markers in older adults [65]. However, supplement intake did not significantly improve overall functional performance, such as the 6-minute walk distance, in clinical trials. These studies support the broader suggestion that microbiome-derived metabolites can affect human muscle-related outcomes. However, these supplementation trials do not establish that exercise-induced microbiota changes mediate improvements in human muscle function.

### 6.1 Distinct but interrelated exercise-related outcomes

Exercise capacity, muscle strength, and physical performance represent distinct but interrelated downstream domains. Exercise capacity represents the integrated cardiovascular, respiratory, metabolic, and muscular ability to sustain activity, commonly assessed via peak oxygen uptake or walking-based tests. However, walking-based measures are also influenced by strength, balance, gait, and motivation; therefore, the boundaries between exercise capacity and physical performance are not absolute [5,60]. Cardiorespiratory fitness has been associated with greater gut microbial richness and higher fecal butyrate concentrations [26]. In older women, improvement in 6-min walk distance following a brisk-walking intervention were positively associated with increases in intestinal Bacteroides, although the nonrandomized design did not establish a microbiome-mediated effect [38]. However, most evidence concerning cardiorespiratory fitness is derived from young or healthy populations rather than frail older adults [26].

Muscle strength, assessed using handgrip, knee-extension, or repetition-maximum testing [5], has shown heterogeneous associations with gut microbiota composition in older adults [60]. Notably, in a randomized trial of women aged 59–79 years, 12 weeks of resistance training increased maximal muscle strength, while no substantial changes in gut microbiota composition were detected [66], indicating that strength gains can occur independently of microbiome alterations.

Physical performance, assessed using gait speed, chair-stand tests, the Timed Up and Go test, or the Short Physical Performance Battery [5,60], integrates balance, coordination, cardiorespiratory capacity, neurological and joint function, and motivation. In the Osteoporotic Fractures in Men cohort of 740 older men, faster 400-m walking speed was associated with greater microbial alpha-diversity, while faster walking speed and a smaller decline in walking speed over approximately 10 years were associated with a higher abundance of taxa with potential short-chain-fatty-acid-producing or anti-inflammatory properties, including *Paraprevotella, Fusicatenibacter*, and *Alistipes* [67]. These findings support an association between the gut microbiome and mobility-related performance but do not establish directionality or causation.

These domains should not be used interchangeably. Aerobic exercise targets exercise capacity, resistance exercise targets muscle strength, and multicomponent exercise targets broader physical performance [68]. The domains overlap substantially, and current evidence does not establish reproducible domain-specific microbiome signatures or demonstrate that microbiome changes mediate improvement in one domain independently of the others [60]. Direct comparative and mediation studies are needed.

### 6.2 Exercise FITT and potential age-related differences

The frequency, intensity, time, and type (FITT) of exercise may influence gut microbiome responses. A systematic review found that microbiome alterations most consistently follow moderate- to high-intensity exercise of 30–90 min at least three times per week, or approximately 150–270 min per week, for eight weeks or longer [69]. However, these values represent patterns observed across heterogeneous studies rather than an established dose–response relationship [69,70].

In studies involving older or late-middle-aged adults evaluating endurance cycling, brisk walking, resistance exercise, and combined aerobic and resistance training over approximately 5–24 weeks yielded inconsistent results [38,66,[71], [72], [73]]. Some reported changes in selected taxa or SCFA [38,[71], [72], [73]], whereas others found minimal or no changes [35,66,[70], [71], [72]]. These studies were generally small and were rarely designed to compare individual frequencies, intensities, session durations, or exercise modalities directly. Consequently, an optimal FITT prescription for modifying the gut microbiota in older adults cannot currently be specified [69,70].

Evidence for age-specific FITT differences remains insufficient. In younger adults, microbiome responses to aerobic exercise differed according to obesity status and reversed after exercise cessation [27], whereas a 24-week concurrent intervention produced only minor compositional changes and no significant changes in alpha- or beta-diversity [74]. In the only study directly comparing age groups, 10 weeks of twice-weekly resistance training increased lean body mass and vastus lateralis thickness but produced minimal changes in gut microbiota composition and fecal or circulating short-chain fatty acids in both younger and comparatively older adults, without a clear age-dependent response [75]. However, the “older” cohort in that study had a mean age of 59 ± 5 years and was not restricted to adults aged ≥65 years; therefore, the findings cannot be directly generalized to geriatric or physically frail populations.

Aging may nevertheless modify microbiome responsiveness through differences in baseline microbial composition, diet, medication use, and overall health status [14,35,45,46,70]. Age-related alterations in intestinal barrier function and chronic inflammation may provide additional biological plausibility [47,49]. Conventional exercise prescriptions for muscle strength, cardiorespiratory endurance, and physical frailty should remain the clinical basis for exercise selection [68], while microbiome-targeted FITT remains a research question [69,70,75].

## 7 Conceptual framework of the exercise–gut–muscle axis

Current findings support the emerging concept of the exercise–gut–muscle axis as a conceptual framework linking physical activity, gut microbiome modulation, skeletal muscle adaptation, physical performance, and frailty trajectories during aging (Fig. 1). In this conceptual framework, exercise acts as a primary physiological stimulus that not only induces direct adaptations in skeletal muscle, but also affects the intestinal environment and microbial ecosystem. Changes in gut microbial composition and metabolic function may subsequently affect the production of metabolites, including SCFAs. These alterations may also affect other signaling molecules that can interact with host metabolic, immune, and mitochondrial pathways related to skeletal muscle maintenance and function. Through these interrelated processes, the gut microbiome links lifestyle habits associated with aging to muscle function and health. Although experimental validation is required in humans, the exercise–gut–muscle axis provides a useful integrative model for understanding how physical activity may influence physical performance trajectories, delay physical frailty progression, and support healthy aging.

### 7.1 Basic assumptions

The proposed exercise–gut–muscle axis is based on three central assumptions.

First, physical activity can alter the composition and metabolic functions of the gut microbiota. Existing observational and intervention studies have shown that exercise is associated with changes in microbial composition and specific microbial taxa, although changes in diversity and the direction of taxonomic responses have been inconsistent.

Second, metabolites and immune signaling pathways derived from the microbiota may influence the physiological function of skeletal muscle. SCFAs and other microbiota-derived metabolites may affect mitochondrial function, inflammation regulation, anabolic responsiveness, and muscle metabolism.

Third, these exercise-induced changes in the gut microbiota may ultimately influence age-related changes in physical performance and frailty trajectories. Therefore, the gut microbiota may function as a biological mediator linking lifestyle to the outcomes of healthy aging.

### 7.2 Predicted relationships

Based on the above-mentioned framework, several biological and clinical relationships can be hypothesized.

Higher levels of physical activity may increase microbial diversity and promote the production of beneficial metabolites such as SCFAs. Increased availability of SCFAs may contribute to the reduction of chronic inflammation, improved metabolic regulation, and enhanced mitochondrial function. These changes may, in turn, lead to improvements in skeletal muscle quality, muscle strength, and physical performance.

From a clinical perspective, this framework suggests that exercise-induced modulation of the microbiome may slow the progression of age-related physical frailty and contribute to the maintenance of functional independence. This hypothesis needs to be confirmed through longitudinal studies and intervention trials.

### 7.3 Scope and limitations

Several factors may influence the proposed exercise–gut–muscle axis.

Dietary habits strongly influence the composition of the gut microbiota and may alter the response to exercise. Medication use, particularly antibiotics and other drugs commonly prescribed to older adults, can substantially alter microbial communities. Polypharmacy, multimorbidity, environmental exposures, and genetic factors may also influence the composition of the microbiome and host responsiveness.

Furthermore, most of the currently available evidence demonstrates associations rather than causality. Therefore, the exercise–gut–muscle axis should be viewed at this stage as a conceptual framework that requires further validation through longitudinal and intervention studies. Furthermore, the available studies rarely distinguished primary from secondary sarcopenia, and most microbiome-related evidence concerns skeletal muscle or physical performance rather than multidimensional frailty. Therefore, the framework should be applied specifically to physical frailty and not be extrapolated to psychological, social, or comprehensive frailty.

## 8 Potential clinical and translational implications

As skeletal muscle weakness and impaired physical performance are central features of frailty, maintaining muscle health remains a priority in geriatric care.

Exercise is one of the most effective interventions for preventing or attenuating frailty [[6], [7], [8], [9]]. In older adults, both resistance and aerobic exercise improve muscle strength, balance, mobility, and physical performance [[7], [8], [9]]. These benefits alone are sufficient to justify exercise as a core component of healthy aging strategies. However, the exercise–gut–muscle axis raises the hypothesis that some benefits of exercise may involve microbiome-related pathways.

Future studies should evaluate whether integrated lifestyle approaches combining exercise, diet, and microbiome-supportive behaviors influence microbiome-related pathways and physical frailty outcomes. For example, sufficient dietary fiber intake may help sustain SCFA production, whereas sufficient protein intake supports muscle protein synthesis and maintenance [17,18,76]. When combined with regular physical activity, these factors may collectively promote a physiological environment conducive to muscle health.

This concept may also support more individualized intervention models in the future. If specific microbiome profiles are found to predict responsiveness to exercise or the risk of frailty progression, microbiome-informed exercise prescription may become possible. However, such applications remain under investigation.

Current evidence suggests that microbiome modulation may represent one pathway contributing to the effects of exercise; however, mediation through the gut microbiome has not been established in humans. However, exercise-based frailty prevention remains a clinical priority. Thus, microbiome-based strategies remain a promising future direction rather than a current standard of care.

## 9 Future research directions

Although growing evidence supports interactions between exercise, the gut microbiome, and skeletal muscle physiology, this field is in its early stages. Therefore, several research priorities should be addressed to advance the exercise–gut–muscle axis from a conceptual model to a clinically actionable framework.

First, many existing studies are cross-sectional, making it difficult to establish causality [26,28,31]. Additional longitudinal studies in humans are needed to better understand how physical activity, gut microbiome alterations, and muscle-related outcomes interact over time. Second, controlled exercise intervention trials in older adults should integrate standardized microbiome and metabolomic assessments with clinically meaningful measures such as muscle strength, gait speed, chair stand performance, and physical frailty status. Third, dietary intake, medication exposure, comorbidity burden, and body composition can markedly affect the microbiome and may confound the interpretation of exercise-related findings [27,30,62,76]. Thus, greater attention should be paid to these factors. Fourth, future studies should determine whether particular microbial taxa or metabolite pathways are associated with improved responsiveness to exercise interventions. Finally, large-scale clinical trials are required to determine whether microbiome-targeted or microbiome-informed strategies can significantly reduce the risk of physical frailty and improve physical function in aging populations. Future studies should prospectively distinguish primary from secondary sarcopenia and compare their microbiota profiles and responses to standardized exercise interventions. Such studies should also determine whether exercise-induced changes improve muscle strength, exercise capacity, physical performance, and physical frailty, and whether these relationships differ by etiology.

## 10 Conclusions

Human observational studies have identified associations among exercise or physical activity, gut microbiota composition and function, skeletal muscle outcomes, and physical frailty. Preclinical and mechanistic studies further suggest that microbial metabolites, intestinal barrier integrity, systemic inflammation, mitochondrial function, and muscle protein metabolism may contribute to these relationships. However, these findings establish biological plausibility rather than confirming a causal pathway in humans. This narrative review highlights that the “exercise–gut–muscle axis” provides a conceptual framework linking physical activity, the regulation of the gut microbiota, skeletal muscle adaptation, and age-related changes in physical performance. Its principal contribution is therefore to provide an integrative heuristic for organizing existing evidence and guiding future research, rather than to offer new causal evidence or a practice-changing clinical model. Clinical intervention studies remain limited by small sample sizes, heterogeneous populations, varied exercise protocols, inconsistent microbiome outcomes, and inadequate control of major confounding factors such as diet, medication use, and comorbidities. Larger, adequately controlled longitudinal studies and randomized trials incorporating standardized exercise protocols, microbiome and metabolite assessments, and clearly defined physical frailty outcomes are needed before microbiome-targeted exercise recommendations can be developed.

## Declarations

### Funding

This work was supported by JSPS KAKENHI Grant Number JP24K02767.

## Ethics approval

Not applicable.

### Consent to participate

Not applicable.

### Consent for publication

Not applicable.

## Data availability

Not applicable.

## Declaration of the use of generative AI and AI-assisted technologies

The authors did not use generative AI or AI-assisted technologies in the preparation of this manuscript.

## CRediT authorship contribution statement

**Shinichiro Morishita:** Writing – review & editing, Writing – original draft, Visualization, Validation, Supervision, Resources, Project administration, Methodology, Investigation, Funding acquisition, Data curation, Conceptualization. **Naoya Iida:** Writing – review & editing, Writing – original draft, Visualization, Investigation, Data curation. **Toshimi Sato:** Writing – review & editing, Writing – original draft, Investigation, Data curation. **Yasuhiro Endo:** Writing – review & editing, Writing – original draft, Investigation, Data curation. **Daiki Tanno:** Writing – review & editing, Writing – original draft, Methodology, Investigation, Data curation. **Shoji Yabuki:** Writing – review & editing, Writing – original draft, Supervision, Conceptualization.

## Declaration of competing interest

The authors declare that they have no known competing financial interests or personal relationships that could have appeared to influence the work reported in this paper.
